# Highly Specialized Ubiquitin-Like Modifications: Shedding Light into the UFM1 Enigma

**DOI:** 10.3390/biom11020255

**Published:** 2021-02-10

**Authors:** Katharina F. Witting, Monique P.C. Mulder

**Affiliations:** Oncode Institute and Department of Cell and Chemical Biology, Leiden University Medical Center LUMC, Einthovenweg 20, 2333 ZC Leiden, The Netherlands

**Keywords:** UFM1, Ubiquitin-like modifiers, substrates, activity-based probes

## Abstract

Post-translational modification with Ubiquitin-like proteins represents a complex signaling language regulating virtually every cellular process. Among these post-translational modifiers is Ubiquitin-fold modifier (UFM1), which is covalently attached to its substrates through the orchestrated action of a dedicated enzymatic cascade. Originally identified to be involved embryonic development, its biological function remains enigmatic. Recent research reveals that UFM1 regulates a variety of cellular events ranging from DNA repair to autophagy and ER stress response implicating its involvement in a variety of diseases. Given the contribution of UFM1 to numerous pathologies, the enzymes of the UFM1 cascade represent attractive targets for pharmacological inhibition. Here we discuss the current understanding of this cryptic post-translational modification especially its contribution to disease as well as expand on the unmet needs of developing chemical and biochemical tools to dissect its role.

## 1. Introduction

Post-translational modification (PTM) with Ubiquitin (Ub) or Ubiquitin-like modifiers (Ubl) such as SUMO, NEDD8, FAT10, ISG15, LC3, and UFM1 governs a plethora of cellular processes ranging from DNA damage response, cell cycle progression, transcription, endocytosis, to proteasomal and lysosomal protein degradation or autophagy [1,2,3,4,5,6,7,8,9,10,11,12]. Ubiquitin fold modifier 1 (UFM1), a small Ubiquitin-like modifier of 85 amino acids (9.9 kDa), is covalently attached to the lysine residues of protein substrates by the virtue of three sequentially acting enzymes. In contrast to other Ubiquitin-like modifiers, UFM1 has long been understudied, the mechanistic and biological details of UFM1 modification are slowly being unraveled.

Highly conserved between species except fungi [13], this small protein modification is structurally similar to Ubiquitin due to the Ub-beta grasp fold but diverges from Ubiquitin and SUMO in terms of sequence, displaying only 21.7% sequence similarity [14,15]. However, the most notable difference is the lack of a C-terminal di-Gly motif, which is replaced by a VG-motif, reminiscent to that of the LC3 family of Ubiquitin-like modifiers. In this light, the enzymatic cascade orchestrating the conjugation of UFM1 to its substrate proteins is unique bearing little semblance to the enzymes of the ubiquitination or SUMOylation cascade positioning UFM1 as a unique and highly specialized PTM [15].

In this review, we provide an overview of the current knowledge on UFM1, its enzymes and substrates with focus on the emerging biological role and contribution to disease. As UFMylation and its underlying biology is starting to attract more attention, we highlight the current assortment of chemical and biochemical tools and its challenges to study this post translational modification.

## 2. The UFM1 (de)Conjugation System

Covalent attachment of Ubiquitin or Ubiquitin-like modifiers to their designated substrate proteins, is orchestrated by the sequential action of three enzyme classes: E1, E2, and E3 enzyme. At the apex of this enzymatic cascade, the E1 enzyme activates the C-terminal glycine residue through adenylation and subsequent thioester formation with its active site cysteine residue, poising it for transfer to the active site cysteine of the E2 enzyme. There the Ub or Ubl is activated again and relayed with the cooperation of the E3 ligase to the lysine residue of the substrate [16]. Additionally, self-modification of Ubiquitin and SUMO through one of their lysines can occur forming polymeric chains that further regulate the biological consequence of substrate modification, which is counter-balanced by the action of specific proteases (i.e., de-ubiquitinating enzymes (DUBs), Sentrin-specific proteases (SENPs), de-Neddylases, or UFM1 specific proteases (UFSPs). While the UFM1 activating and conjugating enzymes share the same concept of ATP-dependent activation and trans-thiolation, their architecture differs greatly from the enzymes of the Ubiquitin and Ubiquitin-like modifiers.

### 2.1. UBA5

**Figure 1:**
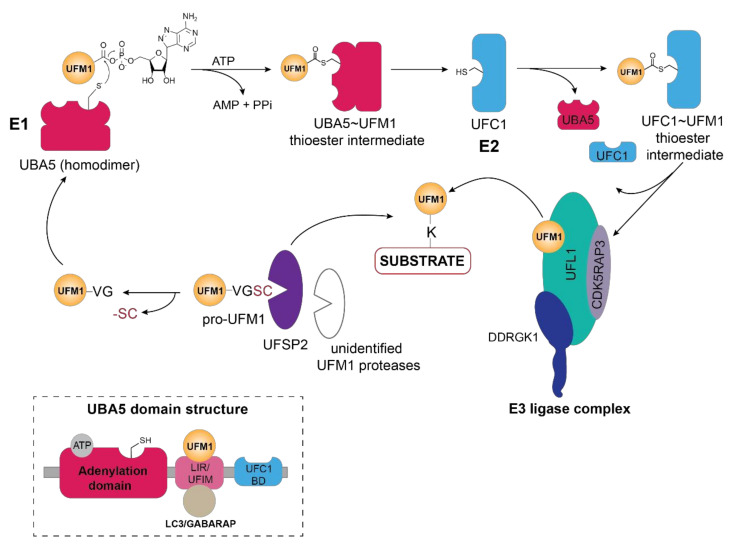
Schematic overview of the UFM1 cascade. In the initial step, the two terminal amino acids (serine and cysteine) are cleaved from pro-UFM1 by UFSP2 and presumably another unidentified UFM1-specific protease [26]. Prior to UFM1 adenylation at the exposed C-terminal glycine residue, UFM1 binds to the UFM1-interacting motif (UFIM)domain of UBA5 (E1), promoting its homodimer formation as well as subsequent UFM1 activation [18]. The UFM1 adenylate intermediate undergoes a nucleophilic attack by the active site cysteine residue of UBA5 yielding the UBA5 ~UFM1 thioester intermediate. Through a transthiolation reaction, activated UFM1 is transferred to the active site cysteine of UFC1 (E2) with the help of the UFC1 binding domain (UFC-BD) of UBA5 [18,19], which then relays the activated UFM1 from the UFC1~UFM1 thioester intermediate via the UFM1 E3 ligase complex to the lysine residue of its substrate proteins. The E3 ligase complex consists of UFL1 and its adaptor proteins DDRGK1 and CDK5RAP3, which stimulate its ligase activity and confer the ER membrane localization of UFL1. Modulation of substrate UFMylation and the resulting cellular response occurs through the action of the UFM1-specific protease UFSP2. Inset depicts the domain organization of UBA5, consisting of the adenylation domain which harbors both the ATP binding pocket and the catalytic site, as well as the LC3-interacting region (LIR) and the UFM1-binding motif (UFIM) and the UFC1-binding domain (UFC1-BD).

Activation of UFM1 through thioester formation by the action of the E1 enzyme (UBA5) at the apex of the enzymatic cascade is key to covalent attachment of this Ubl to its substrates (Figure 1). Firstly, activation of the C-terminal glycine residue occurs through adenylation that then allows the trans-thioesterification through nucleophilic attack of the cysteine residue of the E2 (UFC1) enzyme (Figure 1). Although UBA5, a non-canonical E1 enzyme, lacks a defined active cysteine domain, it contains an adenylation domain harboring the catalytic cysteine [17], reminiscent of Atg7—the E1 enzyme for autophagy-related Ubl LC3—and forms a homodimer through its adenylation domain. Interestingly, UFM1 interaction with the N-terminal UFM1-interacting sequence (UIS) or UFM1-interacting motif (UFIM) adjacent to the adenylation domain of UBA5, is prerequisite to UFM1 activation and subsequent ATP binding as it stabilizes its dimeric state [18,19,20,21] (Figure 1, inset).

**Figure 2:**
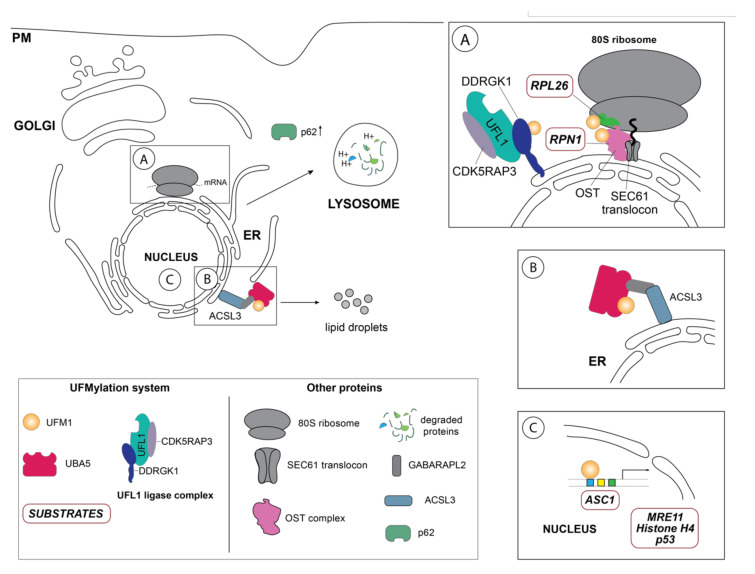
Schematic overview of the so far identified UFM1 substrates and UFM1 enzymes in their respective cellular localization. (**A**) One of the principal UFM1 substrates—the ribosomal protein RPL26—is UFMylated by the orchestrated action of the UFM1 E3 ligase complex consisting of UFL1, CDK5RAP3 and DDRGK1 and contributes to ERAD as well as an alternative ribosomal quality control mechanism following ribosomal stalling at the SEC61 translocon during co-translational protein targeting and ultimately lysosomal degradation of these aberrant proteins [35,36]. The autophagy cargo adaptor p62 (also sequestosome-1 (SQSTM1) has been found in several studies to indirectly regulate autophagy by UFMylation, although the exact mechanistic details are unclear [59]. Additionally, DDRGK1, an adaptor protein recruiting UFL1 to the ER membrane and modulating its ligase activity, is regulated by UFMylation itself and participates in the degradation of ER sheets [38]. Ribophorin I (RPN1), a protein of the oligosaccharyltransferase (OST) complex, has also been reported to be UFMylated [38]. (**B**) Inset depicts the GABARAPL2-mediated interaction between the long-chain-fatty-acid CoA ligase 3 (ACSL3) and UBA5, relocalizing it to the ER membrane and stimulating UFMylation [56]. (**C**) Schematic representation of the nuclear UFMylation substrates, which include the activating signal co-integrator 1 (ASC1), as well as DNA damage repair proteins such as the double strand repair protein MRE11, histone H4, and p53.

Similar to the Ubiquitin-activating enzyme UBE1, human UBA5 has two isoforms differing only by a 56 amino acid long N-terminal extension adjacent to the adenylation domain [21]. Although the short isoform is capable of activating UFM1, biochemical and structural studies have revealed that the N-terminal extension contributes to ATP-binding and modulates the interaction of adenylation domain with ATP such that ATP-gamma phosphate adopts a different structural position compared to the long isoform [20,22,23,24]. In addition to the conformational changes resulting from the lack of the N-terminal region, the catalytic cysteine residue is repositioned on the cross-over loop in the short isoform [21]. Unexpectedly, the presence of the N-terminal region in the long UBA5 isoform increases the ATP binding affinity, resulting in a 1:2 ratio of ATP to UBA5, while the short isoform requires equimolar ATP concentrations [18,20,24]. Prior to relaying UFM1 to the E2 enzyme UFC1, the short C-terminal sequence (UFC1-binding region or UFD domain) of UBA5 recruits the UFM1-thioester intermediate facilitating its adenylation which stimulates trans-thiolation [23] (Figure 1). Moreover, the C-terminal region of UBA5 includes a unique bi-specific LIR/UFIM motif that recognizes both GABARAP and UFM1 through numerous hydrophobic interactions [19] (Figure 1, inset and Figure 2B). Unexpectedly, GABARAP exhibits a ten-fold higher affinity for the LIR/UFIM domain compared to UFM1 and Habisov et al. have reported that its interaction is prerequisite for UBA5 recruitment to the ER membrane [19]. However, GABARAP-mediated translocation of UBA5 to the ER membrane is independent of the lipidation status of this autophagy-related modifier [19,25]. Given that recent research has revealed that the primary UFM1 substrates as well as the E3 ligase UFL1, its adaptor protein DDRGK1 (UFBP1) and the deUFMylase UFSP2 are associated with or anchored in the ER, it is logical that the activating enzyme at the apex of the UFMylation cascade is in proximity. This observation, however, begs the question of how the cytoplasmic E2 enzyme UFC1 translocates to the ER membrane to participate in relaying activated UFM1 to the E3 ligase and ultimately the acceptor lysines of its substrates.

### 2.2. UFC1

Similar to UBA5, the E2-like enzyme UFC1 does not display any conservation with Ubiquitin E2 enzymes and consists of a minimalistic catalytic core domain of 10 amino acids, and an additional N-terminal helix [15,22,27]. Akin to the Ubiquitin E2 enzymes, the active-site cysteine residue catalyzes the trans-thiolation of UFM1 during its transfer from UBA5 (Figure 1). However, structural studies have revealed that the N-terminal helix in UFC1 can adopt a variety of conformations most likely to accommodate different substrates thus conferring substrate specificity [28,29,30]. With predominantly nuclear and partially cytoplasmic localization of the E2-like enzyme UFC1 [15], it is intriguing of whether and how UFC1 localizes to the ER membrane, given that the principal UFMylation substrates and enzymes reside in vicinity of the ER membrane [13].

### 2.3. UFL1

Akin to ubiquitination, the final step of UFM1 activation and transfer onto the lysine residue of the substrate is orchestrated by the E3 enzyme UFL1, also known as RCAD, Maxer, NLBP, and KIAA0776 (Figure 1). However, UFL1 does not display any homology to the HECT or RING domains of the known Ubiquitin ligases, but rather contains a highly conserved N-terminal domain that is instrumental in UFM1 transfer to the substrate [31,32]. Initially discovered by Tatsumi et al., as an interactor of its one of its substrates, UFL1, which is highly conserved throughout the kingdoms, has been demonstrated to catalyze the UFMylation of numerous substrates, most notably UFBP1 (also referred to as DDRGK1, Dashurin, or C20orf116) [32,33]. Later, it was reported that the UFM1 modification of other substrates such as activating signal co-integrator 1 (ASC1) as well as the ribosomal protein RPL26 is mediated by UFL1 [34,35,36] (Figure 2A). The UFM1 ligase UFL1 consists of a transmembrane-like domain flanked by a nuclear localization signal and a primarily through its C-terminal Proteasome, COP9, Initiation factor 3 (PCI) domain, shared by numerous Proteasome subunits, Cop9 subunits, eIF3 translation initiation factor subunits [33,37]. UFL1 does not display any homology to the domains of the Ubiquitin ligases and lacks a catalytic cysteine in its N-terminal domain commonly found in HECT and RBR class of E3 ligases, it seems to function in a scaffolding manner reminiscent of RING E3 ligases recruiting both the E2 enzyme and the substrate [34]. Recruitment of UFL1 to its main venue—the ER membrane—occurs by virtue of DDRGK1 recruitment through its ER signal peptide—as UFL1 lacks a classical transmembrane domain. Concurrently, interaction between UFL1 and DDRGK1 activates its ligase activity, while bringing it in vicinity of its substrates [33,35,38] (Figure 2A). Deletion of DDRGK1 and subsequent reintroduction of variants lacking the transmembrane domain (TM) underscore its role in promoting the proper subcellular localization of UFL1 [33,35,38]. Intriguingly, a substantial pool of UFL1 resides at the ER membrane where it UFMylates several substrates critical in the UPR (unfolded protein response) pathway, however, it is elusive how the nuclear localization signal sequence (NLS) preceding the PCI domain is exposed to translocate the ligase into the nucleus for UFMylation of targets restricted to the nucleus such as ASC1, MRE11, and Histone H4 [34,39,40] (Figure 2C).

Reminiscent of the Nedd8 modification of the Cullin-RING E3 ligases to activate substrate ubiquitination [41], the UFM1 ligase activity of UFL1 requires interaction with its adaptor protein DDRGK1 (also known as UFBP1, Dashurin, or C20orf116). Directed to the ER membrane through its hydrophobic N-terminal signal sequence and anchored by the adjacent transmembrane (TM) domain, the interaction of DDRGK1 with UFL1 as well as the recruitment of the UFM1-specific protease UFSP2 is mediated by the PCI-domain in a UFM1-dependent manner [32,33]. Although originally identified as an interaction partner of UFL1 [33], the highly conserved protein DDRGK1, has also been observed to be modified with UFM1 within the PCI domain at lysine 267 [32,33], which modulates its binding affinity to UFL1 in turn stimulating its ligase activity [34] (Figure 2A)**.** DDRGK1 and in particular its PCI domain mediates the recruitment of UFM1 cascade components —UFL1, C53/LZAP (CDK5RAP3 also known as CDK5 kinase regulatory subunit-associated protein 3), UFSP2, or ACS1—promoting the formation of enzyme-protein complexes fine tune UFM-1 modification [16,32,34].

Consistent with the theme of bringing together multi-component protein complexes, another player participating in the UFMylation pathway is Cdk5rap3 (Cdk5 regulatory subunit-associated protein 3, C53 or LZAP), a highly conserved protein participating in numerous signaling pathways contributing to tumorigenesis and metastasis which is recruited by DDRGK1 [32,42]. Originally identified in the context of DNA damage and cell cycle control [43,44], CDK5RAP3 has been demonstrated to associate with UFL1 as well as DDRGK1 eliciting its subsequent relocalization to the ER membrane [33,45,46,47]. Moreover, complex formation with UFL1 and DDRGK1 as well as CDK5RAP3, promotes its stability by regulating its degradation [47] as well as affecting overall UFMylation as demonstrated by genetic ablation of CDK5RAP3 in mice [48]. Intriguingly, the presence of CDK5RAP3 seems to be requisite for poly-UFMylation [34,35].

### 2.4. UFSP1 and UFSP2

UFMylation of substrates is reversed by the action of UFM1-specific cysteine proteases—UFSP1 and UFSP2 [49,50]. Despite having a catalytic triad universally shared by cysteine proteases, UFSP enzymes lack sequence homology to deubiquitinating enzymes (DUBs) or even other proteases, thus representing a novel subfamily of cysteine proteases [50,51]. UFSP1, has a papain-like fold harboring an unusual active site configuration consisting of both a conserved Cys and His box domain as opposed to the classical Cys-His-Asp catalytical triad [50,51] Intriguingly, while murine UFSP1 processes UFM1 effectively, human UFSP1 seems to be catalytically inactive due to the shorter N-terminus and thus lack of the conserved cysteine active site [49,52]. In contrast, crystal structures reveal that murine UFSP2 contains two domains—a C-terminal catalytic domain with a similar architecture as that of murine UFSP1 in addition to a uniquely structured N-terminal domain key to recruitment by DDRGK1 and subsequent relocalization to the ER membrane [13,51]. UFSP2 predominantly resides in the cytoplasm and the nucleus and requires interaction with DDRGK1 for ER membrane recruitment, insinuating that most of its substrates might be in these cellular compartments and ER localization of this protease is necessitated only in specific cellular contexts, i.e., ER stress, UPR etc. [10]. In contrast to the initial observation by Kang et al. [49] that UFSP2 also cleaves the C-terminal amino acids of UFM1, Ishimura et al. demonstrated that knockout of UFSP2 abolished UFM1 deconjugation from its substrates, resulting in the accumulation of UFMylated proteins [26]. Surprisingly, deletion of UFSP2 did not prevent maturation of pro-UFM1, indicating that the cleavage of the C-terminal amino acids serine and cysteine prior to UFM1 activation, might be mediated by currently unidentified UFM1-specific proteases [15,26,34] (Figure 1).

## 3. Biological Function of UFMylation

Since its discovery more than a decade ago, UFM1 has been linked to diverse cellular processes ranging from hematopoietic cell survival and differentiation during embryogenesis, cell development and tissue homeostasis, transcriptional regulation, mitosis, vesicle trafficking, autophagy, fatty acid metabolism, cellular signaling pathways, and more recently endoplasmic reticulum (ER) homeostasis and DNA damage repair response [16,38,48,53,54,55,56,57]. In contrast to ubiquitination or SUMOylation, UFMylation seems to occur only on several defined substrates [15,33]. Up to date, only a handful of UFM1 substrates including DDRGK1, ASC1, the DNA repair protein MRE11, the ribosomal protein RPL26, ribophorin I (RPN1), and the tumor suppressor protein p53 have been discovered using immunoprecipitation or pulldown approaches (Table 1) [33,34,35,36,38,40,58]. Notwithstanding, the precise molecular mechanisms governing the function of UFM1 on these respective substrates and in the respective cellular pathways are far from being completely understood, as only a handful of UFMylated target proteins have been mechanistically studied up to date (Figure 2).

### 3.1. UFM1—A Matter of Survival during Embryonic Development

Genetic deletion of the UFMylation enzymes UBA5, UFL1, as well as the associated adaptor proteins UFBP1 and CDK5RAP3 in both mice and zebrafish revealed the essentiality of UFMylation enzymes for cellular survival and differentiation, in particular that of hematopoietic stem cells [53,60,61]. Moreover, conditional knockout for these genes in mice invoked apoptosis in hematopoietic stem cells (HSCs) through impairment of primitive and definitive erythropoiesis [10,48,61,62], liver cells, and secretory cells in the intestine (Paneth cells) or the pancreas (pancreatic acini), but also concurrently upregulated genes of the UPR or interconnected downstream pathways including inositol-requiring enzyme 1 (IRE1) and X-box binding protein-1 (XBP1), PKR-like ER protein kinase (PERK), and phosphorylated eIF2a [54,63,64]. In particular, germline deletion of UBA5 in mice invoked in severe anemia ultimately leading to embryonic death, while bone marrow of RCAD^−/−^ mice exhibited defective autophagy, as indicated by increased LC3-II and p62 levels [60,62].

Given that CDK5RAP3 functions as an adaptor protein for UFL1 [48], genetic ablation in zebrafish abolished epiboly, which is the first morphogenic change during the gastrulation stage of embryogenesis, most likely through cell-cycle arrest at the G2/M phase of the cell cycle [65,66]. Moreover, analysis of these CDK5RPAP3 deficient zebrafish revealed defective Wnt/β-catenin signaling, which is critical for normal embryonic development. Similarly, while CDK5RAP3 deficient mice were not viable, conditional knockdown of CDK5RAP3 incited severe hypoglycemia and substantially impaired the lipid metabolism ultimately causing lethality [10,48]. Although the CDK5 kinase plays a key role in diverse cellular processes including cell cycle progression, transcriptional regulation, immunity, apoptosis, senescence, cellular differentiation, and DNA damage response [67], it should be noted that the interaction of CDK5RAP3 with UFL1 largely seems to determine these reported phenotypes, rather than just its association with CDK5 kinase [33,47,48,68,69]. However, whether the interaction of CDK5RAP3 with CDK5 kinase modulates UFL1 recruitment and binding remains to be investigated [48,67].

Moreover, in these mouse embryos, CDK5RAP3 deficiency triggered severe anemia as well as major growth retardation in the developing livers, most likely as a consequence of impaired hematopoiesis [48]. In agreement with the observations that genetic ablation of UBA5, the E3-like ligase UFL1 and its adaptor CDK5RAP3 in mice resulted in defective erythropoiesis [48,60,62], germline deletion of DDRGK1, which functions both as a UFL1 adaptor as well as a substrate, evoked aberrant erythroid differentiation [61]. Further study revealed that in particular the primitive and definitive lineages were affected, while loss of UBA5 was lineage specific affected only the erythroid cell lineages [60,62,63]. Unsurprisingly, genetic ablation of UFBP1, similar to that of other components of the UFM1 system, substantially impedes hematopoiesis and ultimately arrests embryogenesis [61]. In line with these observations, conditional knockout of UFBP1 in mice substantially elevated ER stress, repressed transcription of GATA1 and KLF and affected UFMylation of ASC1, which regulates genes modulating erythrocyte development [10,70]. The observation that DDRGK1 and CDK5RAP3 display a differential effect on the erythroid lineage development seems to be reflective of different substrates or a modulated response to UFMylation. Interestingly, UFMylation of the ribosomal protein RPL26 is also differentially influenced by the action of CDK5AP3 and DDRGK1 [35], indicating that these UFL1 adaptors might recruit other proteins that help shape the cellular response to ER stress [35].

### 3.2. ER Stress: UFM1 to the Rescue

The endoplasmatic reticulum (ER), a dynamic network of membranes is predominantly involved in the assembly and proper folding of proteins in the secretory pathway, which accounts for approximately 30% of the cellular proteome [71,72,73]. To ensure efficiency and fidelity of protein folding, the ER is dynamically regulated by a complex and integrated signal transduction system connecting all components of the secretory pathway. While the ER is constantly adapting to the cellular needs, it becomes essential during ER stress after activation of the unfolded protein response (UPR) to reestablish cellular homeostasis [71,74]. After initiation of the UPR, which utilizes three different signaling pathways (the inositol-requiring transmembrane kinase/endonuclease 1 (IRE1), PKR-like ER protein kinase (PERK), and activating transcription factor 6 (ATF6)), protein translation is halted to allow mRNA translation of UPR proteins, which in turn induce the translation of ER chaperones to upregulate protein folding [71,74]. If this strategy fails to resolve ER stress, the ER network expands and ER-associated degradation (ERAD) of the unfolded or misfolded proteins either by a Ubiquitin-proteasome or by a autophagy-lysosome dependent mechanism as well as upregulating molecular chaperones promoting proper protein folding are initiated [74]. Interestingly, UFM1 was first identified to be transcriptionally upregulated in response to ER stress during the onset and progression of ischemic heart disease in mice [75]. Later, Lemaire et al., observed upregulation of the UFL1 adaptor protein DDRGK1 as well as other components of the UFM1 system upon ER stress induction in the pancreatic acini and the islets of Langerhans [33,76], pointing out the role of UFMylation in secretion [33]. Later, multiple studies also identified the UFL1 adaptor protein DDRGK1 as a UFM1 substrate, requiring UFM1 modification prior to interaction with its cognate ligase [32,33,34,76]. While others have reported UFMylation of DDRGK1 essential to UFL1 ligase function especially during ER stress [34,63], Liang et al., have recently reported that its UFMylation was not required for UFL1 interaction and function during ER-phagy, implying that UFMylation of this adaptor protein might occur only in specific cellular situations and perhaps as a mechanism to regulate its stability or that it might have UFMylation-independent functions [38].

Following the initial observation that the UFM1 ligase UFL1 and its co-factor DDRGK1 modulate ER stress by Lemaire et al., further investigation revealed that transcription of (unfolded protein response) UPR genes such as XBP1 was induced [54] orchestrating the intricate cellular stress response network in a UFM1-dependent manner. Depletion of DDRGK1, which induces moderate ER stress, revealed a regulatory role of the UFM1 system in preserving ER homeostasis through modulation of IREα stability, through its interaction with UFMylated DDRGK1 [63]. Under basal conditions, IREα interacts with BiP (GRP78); however, after UPR activation, it dissociates from BiP, dimerizes and auto-phosphorylates to activate cleavage of the transcription factor XBP-1, which then transcriptionally regulates ER chaperone and ERAD gene expression [77]. Simultaneously, protein expression levels of phosphorylated PERK and BiP (GRP78), key signaling molecules in the UPR-PERK apoptotic pathway, were increased while proteasome inhibition with MG132 prevented Ubiquitin-mediated IREα degradation implying crosstalk with the Ubiquitin Proteasome System [54,63]. Moreover, the intricate interplay between UFMylation and ubiquitination upon disturbance of ER homeostasis becomes evident as genetic disruption of the genes encoding the UFM1 conjugation and deconjugation enzymes, stabilized two soluble luminal ER substrates and integral ER membrane proteins of the HRD1 Ubiquitin ligase [35,78]. In this context, the ubiquitin-dependent degradation of the key UPR signal transducer molecule IREα, which is also a substrate the HRD1 Ubiquitin ligase, is modulated by UFMylation if the interacting protein DDRGK1 [63,77]. Moreover, the action of IREα on the transcription factor XBP-1, introduces an additional regulatory feedback of the UPR response [54,77]. Interestingly, further investigation revealed that impaired UFMylation only had a modest effect on HRD1-dependent ERAD, which may have been causative for the accumulation of misfolded proteins in the ER, as is evidenced by the accumulation of both HRD1 and GRP78 in UFM1 KO cells with ER stress induction [35].

More recently, the repertoire of UFM1 targets has been expanded to ribosomal proteins [35,36,79]. Initially Simsek et al., identified the interaction of UFL1 with the 80S ribosome in a screen for ribosome associating proteins (RAPs) in mouse embryonic fibroblasts as well as the concurrent the UFMylation of three ribosomal subunits—uS3, uS10, uL16, thus implicating a role of UFMylation in embryonic development [79]. Later, Walczak et al., report that RPL26, a ribosomal protein of the large ribosomal 60S subunit located in proximity to the ribosomal tunnel exit is the primary target of UFMylation [35]. Given that UFMylation of RPL26 not only prompts ribosomal ER localization but also appears during translational arrest [36], the authors propose a novel UFMylation-dependent mechanism to resolve translocation-stalled polypeptides and promote the lysosomal clearance of the arrested proteins [36]. In line with this hypothesis, induction of ribosomal stalling by specific inhibitors (i.e., anisomycin) or poly-lysine containing ribosomal stalling constructs (ER_K20), upregulates RPL26 UFMylation thus activating an alternative quality control mechanism distinct from ERAD and cytosolic ribosomal quality control (RQC) to facilitate the lysosomal elimination of these aberrant translation products [80]. Interestingly, the ER stressors, thapsigargin, a sacro/endoplasmatic reticulum Ca^2+^ ATPase (SERCA) inhibitor, and arsenite induced a weaker UFMylation response than translational inhibitors such as ansiomycin, harringtonine, and cycloheximide, implying that RPL26 UFMylation is prompted by inhibition of ribosomal translation [36].

Later, two independent studies identify the ribosomal protein RPL26 as the primary UFMylation target in mammalian cells upon knockout of the deUFMylase UFSP2 [35,36]. In line with the localization of the UFM1 enzymes UFL1, its adaptor protein DDRGK1 and the deUFMylase UFSP2, UFMylation of RPL26 on its two C-terminal lysine residues (K132 and K134) occurs at the ER membrane compartmentalizing these ribosomes [35,36]. Intriguingly, the UFL1 adaptor protein DDRGK1, which is crucial to promoting the localization of both UFL1 and CDK5RAP3, promotes UFMylation of the lysine 134 of RPL26, while experimental evidence suggests that CDK5RAP3 directs the UFMylation of the second lysine residue [35]. In line with the previous findings, Schuren et al., just recently report that the UFMylation pathway modulates the human cytomegalovirus protein US2-mediated degradation of the HLA class I molecule (HLA-I) by ERAD [81]. Similar to Walczak et al., the authors discovered this interaction through a genome-wide CRISPR screen for US2-mediated HLA-I degradation and later identify UFMylated RPL26 by mass spectrometry in a pulldown from HLA-A2-eGFP expressing cells transduced with STREPII-tagged UFM1 [81]. However, the mechanism of how ribosomal UFMylation affects the dislocation of HLA-I from the ER to the cytosol is lacking and requires further research [81]. While numerous studies have linked UFMylation to ER stress, autophagy, and more recently ER-phagy [33,35,36,38,63], it is intriguing that the UFL1-interacting proteins CDK5RAP3 and DDRGK1 seem to play a major role for mediating these biological processes [35,38,63,68].

### 3.3. Autophagy—Self Eating for Survival

One of the cellular strategies to alleviate ER stress in addition to UPR is autophagy in which the damaged proteins or organelles are degraded. In particular, macroautophagy of the ER membrane, referred to as ER-phagy, is utilized by the cell to overcome nutrient depletion by the self-eating of the ER membranes [82]. Given the role of UFMylation in maintain ER homeostasis and in participating in the ER stress response, it is unsurprising that UFM1 is involved in ER-phagy as well [38]. These findings consolidate that UFM1 expression levels are upregulated in response to ER stress while perturbation of the UFMylation system induces UPR, thus connecting UFMylation rather unsurprisingly to various diseases including cancer, type 2 diabetes (T2D), cardiovascular diseases, and alcoholic hepatitis [31,33,75,80,83,84,85].

Upon induction of ER stress through various stressors, the UFM1-specific E3 ligase UFL1 mediates UFMylation of its substrate proteins initiating UPR signaling [31]. Failure to resolve ER stress induces autophagy, a highly selective intracellular degradation process, to remove aberrant proteins or damaged organelles, which are recruited by specific receptors to the expanding phagophore [82,86,87]. Upon formation of the double membrane autophagosome and transport to lytic compartments, the contents is then degraded and recycled. Given that the ER participates in the folding and maturation of nearly 30% of the cell’s proteins [72,73], dedicated quality control mechanisms that are interconnected with cellular degradation mechanisms such as autophagy and proteasomal degradation to ensure the fidelity of the process are critical. Thus, to maintain cellular homeostasis through a properly functioning ER, lysosomal degradation of the endoplasmic reticulum—ER-phagy—can be initiated through highly specific cargo receptors [25,88,89]. While it is well established that the Ubiquitin-like modifier LC3 and ATG8 is the crucial to autophagy and ER-phagy, recent reports now indicate that UFM1 is essential to these cellular degradation pathways [68]. Previously, the marked increase of both LC3-II and the cargo receptor p62 in UFL1 depleted murine bone marrow cells attenuating the autophagic flux linked UFMylation to autophagy [62]. However, while the underlying mechanisms of UFM1 participation in autophagy remained unclear, CRISPR screens identified UFMylation not only as a modulator of p62 expression, but also of the autophagy receptors NDP52 and TAXBP1 upon ER stress induction [59]. While these reports describe the effect of UFMylation on the autophagic pathway, they do not provide mechanistic explanation of how the UFM1 system contributes to this process. Discovery of a dual LC3-like and UFM1 interacting (LIR/UFIM) region in the C-terminus of UBA5, which had originally been identified as an GABARAPL2/GATE-16 interactor [15], provides insights into UBA5 recruitment to the ER membrane to activate the UFL1/DDRGK1 E3 complex within in the ER to accomplish substrate UFMylation [19,25]. Recent research demonstrates that the GABARAPL2-mediated association of UBA5 to the ER depended on the lipid droplet biogenesis factor ACSL3 and thereby possibly facilitating UFMylation dynamics [56] (Figure 2B). Interestingly, depletion of ACSL3 downregulated UBA5, DDRGK1 and UFL1 transcription, suggesting that perhaps transcriptional regulation of UFMylation occurs during lipid droplet biogenesis, which is induced during ER stress and is essential for ER-phagy [38,90]. Just recently, C53 (CDK5RAP3), was identified in an immunoprecipitation-mass spec (IP-MS) screen as a receptor for proteins destined for autophagic degradation [68]. Further mechanistic dissection revealed that C53 interacted upon stalled proteins upon ribosomal stalling during co-translational protein translocation and its depletion diminished autophagic degradation of these aberrant proteins [68].

Corroborating these observations, Jiang et al., report that DDRGK1-mediated UFMylation of RPL26, which mediates the degradation of ER sheets, as well as of the quality-control factor ribophorin 1 (RPN1) glycosylating faulty ER proteins during co-translational translocation [38,89]. Perhaps unsurprisingly in the context of ER-phagy, numerous proteins involved in endocytic pathway the RAB-GTPases RAB1A/RAB5C, Clathrin, and the ADP-ribosylation factor ARF4 were identified to be UFMylated in a DDRGK1-dependent manner [38]. Earlier, Zhang et al., found that perturbation of ER homeostasis but also inhibition of vesicle trafficking with Brefeldin A upregulated the expression of UFM1, UBA5, UFL1 and C53, which could be reversed by depletion of XBP-1 [54]. In line with these observations, UFC1 and UFM1, were identified to interact with the neural adhesion molecule NCAM 140 (also known as NCAM1, isoform 2 or CD56) promoting its endocytosis, implicating UFMylation in the endocytic pathway [91]. In addition to regulating ER homeostasis primarily through post-translational modification as well as through protein interaction with DDRGK1, UFM1 has been implicated in transcriptional control of several genes involved in the cellular UPR. Most notably, p62 (SQSTM) and XBP1 have been demonstrated to be transcriptionally modulated by UFMylation introducing an additional layer of ER homeostasis maintenance [54,59]. Genetic depletion of UFM1 induced CHOP and SQSTM expression, while Zhang et al. report that UFM1 binds to XBP-1, a transcription factor crucial to initiating the UPR response as well as a plethora of dedicated transcriptional networks regulating cell-type and condition-specific responses, in ChIP and luciferase assays [54].

### 3.4. Transcriptional Regulation

While UFM1 has been shown to transcriptionally regulate UPR to restore cellular homeostasis [54,63], it has also been linked to transactivation of estrogen receptor α (ERα) as well as in modulating expression levels of genes critical for erythropoiesis [53,61]. Moreover, the transcriptional activation of activating signal co-integrator 1 (ASC1) is induced by UFMylation to promote estrogen receptor α (ERα) transactivation [34]. One of the few identified targets is the activating signal co-integrator (ASC1), a known co-activator of the estrogen receptor ERα, was discovered to be UFMylated in a DDRGK1 dependent manner at four different lysine acceptor sites (K234, K325, K334 and K367) [34]. Interestingly, UFMylation of ASC1 occurred only upon stimulation with estrogen (E2), which displaced UFSP2 bound to ASC1, enlisting UFL1 and DDRGK1 to promote poly-UFM1 chain formation by utilizing the internal lysine (K69) of UFM1 [34]. UFMylation of ASC1 is key to promoting ERα transactivation as well as transcriptional activation of ERα target genes such as pS2, cyclin D1, and c-Myc [34]. These findings indicate that UFMylation of ASC1 might mediate tumor formation and growth in ERα-dependent breast cancer [34]. Correspondently, Zhang et al., observed that the expression levels of genes regulating erythroid lineage (ε-globin, βH1-globin) and the cognate transcription factors (GATA-1/FOG-1 and KLF) were markedly reduced in UFL1 deficient fetal livers, thus assigning UFM1 a role in transcription [62]. In addition to partaking in transcriptional regulation of a variety of genes [92], the long non-coding RNAs (lncRNA) of UFC1, which are large RNA transcripts that do not encode proteins but have regulatory roles [92,93], have been found to critically modulate oncogene expression thus contributing to tumorigenesis, progression and metastasis, in numerous cancers [94,95].

### 3.5. Signaling Pathways

Corroborating its role in modulating transcription, UFM1 has been implicated in orchestrating cellular signal transduction pathways such as the NF-kB and JNK signaling cascades [83,96,97]. Initial studies described that both UFL1 and the adaptor protein C53 (CDK5RAP3), reported to function as a tumor suppressor [43,98,99,100], regulates NF-kB signaling by associating directly with RELA (p65), abolishing its phosphorylation to enhance interaction with HDAC thereby stimulating NF-κb activity [47]. Later, repression of liposaccharide (LPS) induced nuclear translocation of NF-κb, which initiates its transcriptional activation as well as transcription of inflammatory genes such as TNF-α, IL-6, IL-1β, and IL-12, was found to be modulated by UFM1 system [84,96,101]. Another example of the role of UFM1 in coordinating cellular signal transduction is the UFMylation of ASC1 upon ERα binding promoting the ERα transactivation to modulate expression of ERα target genes including Cyclin D1 and c-Myc [34]. In the light of these findings, it becomes clear that UFMylation is an important player in signal transduction, however many details and the extent of its participation remain to be understood.

### 3.6. UFMylation and the DNA Damage Response

Upon insult to the genome leading to DNA double strand breaks (DSBs), expeditious DNA damage response (DDR) is required to maintain genomic integrity and is tightly regulated by a host of post-translational modifications including phosphorylation, methylation, acetylation, ubiquitination, and more recently UFMylation [102]. Initiation of DDR occurs through activation of the MRE11-RAD50-NBS1 (MRN) complex subsequent to histone H2AX phosphorylation at the DNA damage sites mediated by ataxia-telangiectasia mutated (ATM) kinase leading to DNA repair protein recruitment by the MRN complex [103]. Moreover, further regulation of ATM is introduced by the interaction of the acetyltransferase Tip60 with tri-methylated histone H3 (H3K9me3) which then induces ATM acteylation thus amplifying its activation through its autophosphorylation [103]. However, UFL1-mediated UFMylation of MRE11 on lysine 282 was found to be critical for MRN complex formation, immediate recruitment to DNA damage sites, as well as for proper ATM activation to initiate DNA repair [40]. Prior to activation and recruitment of the MRN complex to the DNA double strand breaks (DSBs), ATM kinase phosphorylates UFL1, an interactor of the MRN complex, enhancing its ligase activity and inciting UFMylation of histone H4 in a Tip60-dependent manner, providing a positive feedback loop for ATM activation [39]. Furthermore, Qin et al., found that UFL1-mediated histone H4 mono-UFMylation, coordinates Tip60 and Suv39h1 recruitment in a serine/threonine kinase 38 (STK38) dependent fashion and mediates subsequent histone H3 trimethylation (H3K9me3) to synchronize the DDR [39,104]. Just recently, the tumor suppressor p53, which is post-translationally modified by phosphorylation, acetylation, SUMOylation, NEDDylation, and ubiquitination orchestrating a differential cellular response to a plethora of cellular stimuli [105], was reported to be UFMylated at four lysine residues in C-terminal domain intrinsically suppressing tumorigenesis [58]. UFMylation at this PTM hotspot, possibly prevents MDM2 ligase recruitment and subsequent ubiquitination thereby antagonizing p53 degradation. Despite this finding, further research needs to be undertaken to investigate the crosstalk between UFMylation and other PTMs on p53 in response to cellular stressors or DNA damage.

In the light of earlier findings that UFM1 modification is necessitated for timely DNA damage response upon double strand breaks [11,39,40,104], UFMylation of p53 could potentially represent a signalling pathway leading to the rapid MRN complex recruitment and concomitant activation of ATM kinase [39,40,58]. The participation of UFM1 in the early events of the DNA damage response pathway as well as its role in modulating p53 function, links the Ubiquitin-like modifier with disease onset, in particular with tumorigenesis and cancer progression.

## 4. UFMylation and Disease

Given that UFMylation has been associated with numerous biological processes, most notably ER stress and maintaining protein homeostasis [10], the UFM1 system is a potential key player in the pathogenesis and progression of numerous diseases ranging from diabetes to cancer, a range of inflammatory diseases, certain hepatitis types, and neurodevelopmental disorders [75,80,106,107,108,109,110,111,112,113]. Although the discovery that the ribosomal protein RPL26 as a predominant UFMylation substrate had shed insights into the role of UFM1 in maintaining ER homeostasis (Figure 1) [35,36,38], it also establishes the contribution of the UFM1 system to a variety of diseases arising from disturbance of ER homeostasis ranging from cardiac disease, gut inflammation, and cancer [64,75,80,84,98], thus warranting further study to fully understand its physiological role.

Recent studies have demonstrated a protective role for UFMylation against cardiac myopathy, as UFL1 depletion in cardiomyocytes, which induces ER stress, led to increased fibrosis, decreased cardiac contractility and hypertrophy under pressure overload compared to the controls [84]. In line with the biological function of UFL1 and its activator DDRGK1, these UFMylation proteins safeguard from the effects of prolonged ER stress in pancreatic beta-cells and other endo- and exocrine cells and thus possibly contributing to the development of type 2 diabetes, steatohepatitis, pancreatitis, ischemic heart injury, as well inflammatory bowel disease [33,64,75,83,113,114]. Moreover, UFM1 possibly plays a contribution to tumorigenesis, as demonstrated by the UFMylation of ASC1 and the subsequent transactivation of the ERα [34] and its role in PI3K signaling in gastric cancer [57].

Of note is that the expression of long non-coding RNA (lncRNA) and long intergenic RNA (lincRNA) for UFC1 regulates the progression of numerous cancer types including gastric, breast, pancreatic and colorectal cancers through modulation of specific micro-RNAs [94,115,116,117]. However, the mechanistic basis of these RNA transcripts containing UFC1 is far from clear and requires more research to unravel how this affects UFMylation and cellular homeostasis. Nonetheless, while some association between the UFM1 system and diseases such as cancer or diabetes could be established, many disorders caused by dysfunctional UFM1 enzymes were discovered to be of genetic origin. For example, genetic screening revealed the participation of UFM1 enzymes in several neurodevelopmental disorders ranging from infantile encephalopathy, microencephaly, and ataxia [106,109,110,111,112,118,119,120,121]. Interestingly, the enzymatic function of UBA5 or UFC1 was compromised due to loss-of-function mutations or because of a polymorphism in the UFM1 promoter, thereby attenuating UFMylation [108,109,118]. Despite these relevant findings, it remains enigmatic how UFMylation contributes to the onset of these pathologies. Continuing with this theme, familial dominant mutations in the UFSP2 or DDRGK1 gene resulted in Beukes and Sohat hip dysplasia, with loss of DDRGK1 function abolishing SOX9 mediated transcription of type II collagen [122,123,124].

## 5. Tools to Study UFMylation

Given that the UFM1 system is structurally and functionally different than that of Ubiquitin and Ubiquitin-like modifiers, dedicated tools are urgently needed to study the UFM1 enzyme cascade as well as its substrates. Although a few biochemical tools have been developed in past years, the field urgently requires innovative activity-based probes and proteomic approaches to fully profile UFM1 enzymes and substrates.

### 5.1. Identifying UFM1 Substrates—The Key to Unraveling UFM1 Biology

Initially discovery of UFM1 and its conjugating enzymes occurred through immunoprecipitation through specifically generated antibodies for affinity pulldown approaches [15,33,49]. Similarly, the identification of the E3 ligase UFL1 and its substrate DDRGK1 was by the virtue of these biochemical methods [33]. Similarly, other UFM1 substrate proteins such as ASC1, MRE11, and Histone H4 were discovered primarily by affinity pulldowns [33,34,39,40] (Table 1). A first attempt to systematically unearth substrates modified by Ubiquitin-like modifications (Ubls) including UFM1 has been described in a study conducted by Pirone et al., in which the corresponding E2-E3 enzymes in conjunction with the biotin-tagged Ubls are ectopically overexpressed [125]. Although this approach enabled the identification of a few potential UFM1 substrates such as the cytochrome B5 reductase 3 (CYB5R3), the proteasomal subunit PSMB5 and the DNA double-strand break repair protein MRE11, it suffers from numerous limitations ranging from the use of only one cell line, the absence of specific external stimuli (i.e., cellular stress etc.) as well as the exogenous overexpression of the E2 and E3 enzymes, which might alter the expression levels of the target proteins or result in overexpression-induced artefacts. Moreover, the UFM1 targets identified in this study were primarily localized in the nucleus, which is unsurprising given that the cognate E2 (UFC1) was concomitantly overexpressed [125].

More recently, an improved yeast-two-hybrid interactome screening methodology utilizing the ligases as baits to identify interaction partners followed by expression of the enzymes required for PTM modification of the substrates in *E. coli* [126]. Identification of the PTM modification sites on the substrates by proteomics as well as validation of the substrates in vitro and in cells permitted the discovery of bona fide substrates [126]. Using this approach, sixteen UFL1 interacting proteins could be identified and two novel UFL1 substrates—Metallothionein (MT1M) and TSC22D3—were demonstrated to be UFMylated [126].

**Table 1:** Overview of methods and conditions used to identify UFM1 substrates and interacting proteins.

Method	Constructs and Experimental Conditions	Substrates or Interacting Proteins	Reference
Affinity purification	Strep-Tag UFM1 overexpressed in insulin-producing MIN6 cells	UBA5, UFC1, UFL1, DDRGK1	[33]
	GST-UFM1		
	Flag-His-UFM1	ASC1	[34]
	Flag-UFL1 and DDRGK1	p53	[58]
	His_6_-UFM1ΔSC	RPL26 and RPL26L1	[35,36]
	Flag-UFM1 (WT and ΔC3) in UFSP2 CRISPR knockout cells and mass spec		
	Flag-DDRGK1	ANT3 ^1^, NUP160 ^1^, and SF3B1 ^1^ (DDRGK1 associated proteins)	[76]
	BirA^OPT^-UFM1-UFC1 vector	20 UFM1 substrates/interactors found incl. MRE11. Only CYB5R3 and PSMB5 were validated	[125]
	StrepII-tagged UFM1	RPL26, RPS3 ^1^, RPS20 ^1^, RPL10 ^1^ and several UFM1 interactors	[81]
Yeast two-hybrid screening		Originally GATE16 was discovered as a UBA5 interactor, and later identification of UFM1 by AP-MS	[15]
Yeast two hybrid and PTM reconstitution in *E. coli*	pDEST32 bait and prey vectors; pYESS-PIP and pYESS^PTM^ vectors	16 potential substrates or interactors; only DDRGK1, MT1M^1^, and TSC22D3 ^1^ were validated	[126]
Co-immunoprecipitation and microscopy experiments	His-UFM1 pulldown under denaturing conditions after induction of DNA damage followed by MS/MS	Histone H4 and three other potential substrates (ZNF281 ^1^, ZIC2 ^1^, and CAPNS1 ^1^)	[39]
	Colocalization of UFL1 and y-H2AX upon irradiation; co-immunoprecipitation of HA-UFL1 revealed interaction with MRE11	MRE11	[40]
Protein microarray and ELISA	GST-NCAM140 (cytoplasmic domain)	UFC1 interaction with the cytoplasmic domain of NCAM 140	[91]
Protein microarray	Profiling of Ubl modification of about 9000 proteins before and after release from mitotic arrest	Identification of 202 UFM1 targets (transmembrane transporters, ion channels, cytokine transporters); hit validation required	[55]
Peptide array	UBA5 peptides and GST-LC3 and GST-GABARAP	Mapping of LIR/UFIM motif in the C-terminal domain of UBA5	[19]
Peptide-competition coupled affinity proteomics screen	IP-MS screen under ER stress conditions using a synthetic ATG8-interacting motif peptide with higher affinity than ATG8	C53 (or CDK5RAP3) was revealed to bind ATG8	[68]
CRISPR screens	CRISPR knockout screens (CRISPRi)	p62 (SQSTM), DDRGK1, RPN1	[38,59]
	CRISPR-mediated HA-tagging of endogenous proteins (hATG8)	ACSL3	[56]
Chromatin Co-immunoprecipitation (ChIP)	Endogenous and overexpressed XBP-1s (Flag-XBP-1s)	XBP-1s binds to the promoter region of UFM1	[54]
Bioinformatic analysis	Analysis of proteins with UFM1 binding motif [19]	STK38 (NDR1) binding to UFMylated Histone H4 in complex with KAP-1 ^1^ and SUV39H1	[104]

^1^ ANT3 (ADP/ATP translocase 3); NUP160 (nuclear pore complex protein Nup160); SF3B (splicing factor 3B subunit 6); RPS3 (40S ribosomal protein S3); RPS20 (40S ribosomal protein S20); RPL10 (60S ribosomal protein L10); MT1M (Metallothionein-1M); TSC22D3 (TSC22 domain family protein 3); ZNF281 (zinc finger protein 281); ZIC2 (zinc finger protein ZIC2); CAPNS1 (calpain small subunit 1); KAP-1 (also known as TRIM28 or transcription intermediary factor 1 beta).

Notwithstanding that these biochemical approaches especially in combination with genetic methods (i.e., CRISPR screens) are powerful tools for identifying novel enzymes, these methods typically rely on the ectopic overexpression of UFMylation enzymes to enhance UFMylation of its substrates [32,33,34,35,36]. Consequently, these approaches suffer from numerous limitations including the introduction of overexpression artefacts (i.e., mislocalization, protein aggregation), the alteration of the stoichiometry of the individual components, induction of unwanted cellular responses (i.e., ER stress, activation of signaling pathways, effects), transcriptional activation or deactivation [127,128,129,130]. In particular, the identification of UFMylated substrates has proven challenging employing traditional affinity-purification mass spectrometry (AP-MS) techniques, and the lack of antibodies analogous to the di-Gly antibodies employed for detecting the Ubiquitome [131], the development of innovative affinity pulldown and proteomics methodologies is warranted.

### 5.2. Towards Development of a UFM1 Toolkit Enabling Inhibitor Discovery

The first activity-based probe—Flag-UFM1-VME—was generated using intein chemistry and permitted the identification of the UFM1-reactive cysteine protease UFSP1 from mouse spleen lysates [49]. With the UFSP1 sequence in hand, murine UFSP2, another UFM1 reactive protease, which contains a longer N-terminal extension, could be uncovered by sequence comparison [49]. Later, full synthetic strategies were developed, either via sequential α-ketoacid-hydroxylamine (KAHA) ligations [132] or native chemical ligation [52], that made the facile synthesis of C-terminal variants possible. Given the advantages of peptide synthesis to generate UFM1, specialized reactive groups such as propargyl-amine or dehydrolalanine as well as any tag for detection or retrieval could easily be incorporated, facilitating an array of biochemical and biological experiments [52]. Given the emerging importance of the UFM1 system in a variety of diseases, access to facile synthesis methods would also permit the expansion of assay reagents to screen for inhibitors for the UFM1 enzymes. Initial attempts at developing UBA5 inhibitors have been undertaken utilizing adenosine moieties [23,133] or a ligand-based approach [134], however, these compounds require substantial improvement to attain efficacy and warrant clinical application. In line with the participation of UFMylation system in hematopoiesis, Chen et al., reports that salubrinal, initially identified as an eIF2α dephosphorylation inhibitor preventing ER stress induced apoptosis, also targets the UFMylation pathway [64,135]. While this mechanism is likely to be indirect and not by direct inhibition of UFM1 enzymes, reversal of DDRGK1-depletion induced anemia could be observed [135]. However, despite the considerable recent progress in developing new chemical tools and innovative proteomic approaches, systematic approaches to efficiently identify UFM1 substrates in diverse cellular contexts are urgently needed.

## 6. Outlook and Discussion

From its discovery more than a decade ago, not only the conjugating and deconjugating enzymes of UFM1 have been identified and characterized, but also its biological function is starting to be unraveled. However, in contrast to Ubiquitin and other Ubiquitin-like modifiers, UFM1 predominantly occurs as a single modification on lysine residues of its targets with two central themes—mediating protein-protein interactions and regulation of the transcription of specific proteins in a given cellular pathway. While the enzymes mediating the covalent attachment of UFM1 to the lysine residues of its target proteins have been identified and biochemically characterized, many questions regarding mechanistic details remain unanswered. While UBA5 had been mechanistically and structurally extensively studied [18,21,22,23,24], the E3 ligase UFL1 has not been mechanistically dissected or structurally characterized. Given that UFL1—the only E3 ligase identified so far—bears no semblance to either HECT or RING domain E3 enzymes, obtaining and solving its structure will not only unlock its unique mechanism, but also aid the discovery of other potential UFM1 E3 ligases through bioinformatic approaches. Additionally, identifying the substrates of UFL1 by utilizing novel methods such as the TULIP2-methodology [136,137], which has been successfully employed to uncover SUMOylated substrates of the STUbL RNF4 ligase, would shed enormous insight into UFM1 biology.

Localization of the UFM1 enzymes and their adaptor proteins provides indications as to the nature of its substrates and biological pathways. While Huber et al., recently reported that UBA5 association with GABARAPL2 relocates UBA5 from the cytoplasm to the ER membrane in close proximity to the E3 ligase and its substrate proteins [25], it remains elusive of whether and how the E2 enzyme UFC1 is recruited to this organelle. Interestingly, UBA5 is predominantly localized in the cytoplasm, and only a small fraction is recruited to the ER, indicating that the majority of the UFM1 substrates are perhaps cytoplasmic and participating in other cellular processes. Although UFL1 contains a nuclear localization signal sequence, it is unclear how this is exposed to facilitate nuclear translocation of UFL1 and how the other UFMylation enzymes are recruited in order to UFMylate proteins such as ASC1, p53, histone H4, or MRE11. However, despite recent discoveries of UFMylated proteins, detection of more substrates remains challenging due to the low abundance of UFM1, the highly transient nature of UFMylation necessitating suitable knockout strategies [26], the lack of antibodies recognizing the C-terminal VG-motif of UFM1 coupled with innovative proteomics methodologies. By contrast, bioinformatic analysis has enabled the prediction and later discovery of a many Ubiquitin enzymes and ligases [138,139,140,141], but this remains problematic since the UFM1 enzymes exhibit a low degree of conservation to the enzymes of the Ubiquitin cascade [15], warranting the introduction of innovative proteomic and biochemical approaches in conjunction with advanced bioinformatic techniques to unravel the mysteries of the UFM1 conjugation system. Moreover, advancement of innovative chemical tools with specialized reactive groups catering to the unique architecture of the UFM1 enzymes is urgently required in order to facilitate the discovery and characterization of novel enzymes but also to expedite inhibitor development. Although a few compounds have been reported to inhibit the activity of the E1 enzyme UBA5 [23,133,134], progress has been hampered due to lack of suitable assay reagents necessitating their introduction.

Since its discovery, the UFM1 system has been demonstrated to participate in a numerous cellular pathways, most notably in maintaining ER homeostasis via a non-canonical quality control mechanism, DNA damage response coordination, scaffolding large protein complexes, and contributing to tumor suppressor functions [16,35,36,38,43,58,142]. With UFMylation modulating these cellular responses, it is unsurprising that dysregulation of this PTM inevitably leads to diseases such as cancer, diabetes, and inflammatory processes [13,64,114,142]. However, the underlying biology of UFMylation needs to be unraveled and fully understood in the context of pathogenesis employing innovative chemical tools and biochemical approaches to shed light into this enigma and pave the way for novel therapeutic approaches.
